# 

**Published:** 1885-12

**Authors:** 


TO THE

### INDEPENDENT PRACTITIONER.

### VOLUME VI.

ORIGINAL COMMUNICATIONS.

Action of Salicylic Acid Upon Teeth, The. Chas. Mayr................ 245
Adapting of Artificial Dentures. C. H. Land......................... 12
Address of Welcome. L. A. Thurber................................... 364
Amalgams. W. St. Geo. Elliott....................................... 62
Amalgam Solvents. W. D. Miller...................................... 527
Anæsthetic, A New. G. L. Curtiss.................................... 22
Annual Dinner of the American Dental Society of Europe, The. A Member 578
Anticipating Caries. C. E. Francis.................................. 574
Atmospheric Pressure in the Retention of Entire Dentures. W. B. Ames.. 347

Biological Studies on the Fungi of the Human Mouth. W. D. Miller..227, 283

Case in Practice, A. W. D. Miller................................... 19
Cervical Edge, The. J. Smith Dodge, Jr.............................. 584
Child's Teeth, A. W. Geo. Beers..................................... 73
Cocaine Hydro-Chlorate. A. M. Ross.................................. 131
Cocaine in Dental Surgery. Theo. Hillischer......................... 416
Clinic of Prof. Brophy. H. L. Barnum................................ 251
Clinic of Prof. Garretson. Robert S. Ivy.......................249, 580
Cocaine in Operation for Cleft Palate. Roswell Park................. 135
Cohesive Gold. J. Smith Dodge, Jr................................... 69
Combination of Napkin and Rubber Dam. B. M. Wilkerson............... 21
Comprehensive Degrees. C. E. H. Phillips............................ 306
Comma Bacillus in the Human Mouth, A. W. D. Miller.................. 246
Comma Bacillus of Asiatic Cholera, The. Geo. W. Lewis............... 178
Construction of Suersen's Obturator. Theo. Weber........... 188, 243, 290
Critical Review, A. C. H. Land...................................... 468
Crown Work. A. M. Ross.............................................. 9

Das Zahnærztliche Institut. W. D. Miller............................ 459
Dens Sapientiæ. J. W. Clowes........................................ 190
Dental Encyclopœdia. B. R. McGregor................................. 76
Dental Hygiene. G. L. Curtiss....................................... 302

Dental Nutrition. J. R. Walker.......................... 569
Dental Jurisprudence. J. Allen Osmun.......................... 123
Dental Profession of England, The. Felix Weiss.......................... 470
Dental Societies and Dental Literature. C. J. Ellis.......................... 358
Dental Suggestions. John D. Wingate.......................... 305
Dentistry in France. K. Holms.......................... 351
Discussion of Some Questions in Dental Caries, A. W. D. Miller.......... 171
Diseases of the Period of Dentition. W. C. Barrett.......................... 623

Earthy Phosphates, The. W. C. Barrett.......................... 453
Editorial Responsibility. Geo. H. Cushing.......................... 643
Effects of Zymotic Diseases Upon the Teeth, The. F. S. Whitslar....... 462
Electric Lamp, The. W. H. Trueman.......................... 133
English Tube Teeth. Lawrence Vanderpant.......................... 582
Epithelioma. A. M. Ross.......................... 232

Facilitation of Contour Work. E. A. Bogue.......................... 128

Gastrotomy for the Removal of an Artificial Denture. W. D. Miller ..... 520
Gold and Tin Combined as a Filling Material. A. W. Harlan............ 565
Gum-Chewing Variety, A. C. M. Wright.......................... 361

Herbst Method of Filling Teeth, The. C. F. W. Bodecker.............. 395

Materials for Finishing and Polishing Fillings. C. F. W. Bodecker...... 640
Mechanical Aids in the Treatment of Pocket Diseases of the Alveolus. J. N. Farrar.......................... 509
Menthol in Dentistry. C. E. H. Phillips.......................... 422
Method of Capping Exposed Pulps, A. W. M. Ramsdell................ 71
Mirabile Dictu. C. E. Francis.......................... 75
More Prehistoric Dentistry. W. H. Waite.......................... 195
Muriate of Cocaine. W. D. Miller.......................... 192

New Anæsthetic, The. Julien W. Russell.......................... 16
New Comma Bacillus, A. W. D. Miller.......................... 471

Obturators and Artificial Vela. N. W. Kingsley.......................... 304
On the Availabilityof Certain Antiseptics in the Prophylactic Treatment of the Oral Cavity. W. D. Miller.......................... 410

Painless Removal of Living Pulps, The. G. M. Merritt.................. 195
Practical Experience in the Use of Oxy-Phosphate Cements. W. D. Miller. 59
Practitioner and Instructor. C. E. H. Phillips.......................... 194
Proliferation of Epithelium in Alveolar Abscess. W. D. Miller........... 5

Relation of Hygiene to Practical Dentistry. C. E. H. Phillips........... 586
Repairing Vulcanite Work. S. B. Palmer.......................... 18
Reply to Dr. C. H. Land's Critical Review. W. B. Ames.................. 522
Reproduction of Tissues by Sponge Grafting. W. H. Atkinson........... 465
Rough Enamel. C. E. Francis.......................... 20

Scalers and Root Trimmers. E. S. Talbot........................ 226
Some Evidences of Prehistoric Dentistry. J. G. Van Marter............ 1
Some of the Pathological Conditions of the Oral Cavity. F. D. Nelles.... 513
Some Points in the Application of Hydro-Chlorate of Cocaine to Dental Surgery. N. J. Hepburn........................................ 7
Some Recent Events Connected with the Dental Profession in England. W. H. Waite........................................ 339
Standard of Work on the Natural Teeth that the Dentist of the Future Must be Equal to E. Parmly Brown ........................... 66
Straining at a Gnat. C. E. Francis........................... 238
Study of Tooth Development with Special Reference to the Formation of Enamel, A. Henry Beates........................................ 296
Success with Cocaine, A. J. Smith Dodge, Jr........................ 196

Teeth of Deaf Mutes, The. J. Howard Reed........................ 419
Too Little of a Good Thing. J. G. Templeton........................ 517
Treatment of Defective Teeth During Pregnancy. C. E. Marvin......... 115

Use of Cocaine by Hypodermic Injection for Extraction of Teeth, The. D. W. Barker........................................ 300
Use of English Tube Teeth, The. Lawrence Vanderpant................ 524

World's Exposition, The. "Mrs. M. W. J."........................ 148

### REPORTS OF SOCIETIES.

American Dental Association.................. 23, 472, 528, 587, 644
American Dental Society of Europe........................79, 596
American Medical Association........................323, 372
Annual Dinner of the New York Odontological Society................ 152
Association of Dental College Faculties........................ 491

Central Dental Association of Northern New Jersey................86, 137
Chicago Dental Society........................37, 93, 148

Dental Society of the State of New York........................315, 429

Fifth District Dental Society........................ 269

Massachusetts Dental Society........................ 45
Michigan State Dental Association........................ 313
Mississippi Valley Dental Association........................197, 253

National Association of Dental Examiners........................ 497

Odontological Society of New York..........42, 95, 204, 331, 379, 605, 661

Pennsylvania State Dental Society........................31, 487, 539

Southern Dental Association..................265, 307, 365, 423

The Section of Oral and Dental Surgery in the International Medical Congress........................................ 655

### EDITORIALS.

Case in Practice, A........ 209
Case of General Grant, The........ 210
Chicago Annual Reunion........ 48
Close of the Volume........ 673
Cocaine........ 49
Cocaine in Dental Practice........ 207
Cocaine in Dentistry........ 558
Comma Bacillus........ 274

Deferred........ 388
Dental Congress, A........ 559
Dental Diseases in Animals........ 333
Dental Histology........ 158
Dental Section of the International Congress, The........ 671
Dental Section of the International Medical Congress, The........ 156
Dental Surgery........ 385
Diploma Traffic, The........ 49
Doctor W. H. Dwinelle........ 159

Editors and Editing........ 668
Editorial Responsibilities........ 554

First District Dental Society of the State of New York........ 613

General Grant's Condition........ 218
Gum Chewing........ 386

Herbst Method of Filling Teeth, The........ 270

Important New Departure, An........ 503
Index Medicus........ 274
International Medical Congress........ 100

Japanese Dentists........ 387

Matrices........ 98
Medical Congress........ 557
Medical Congress of 1887, The........ 437
Meeting at Minneapolis, The........ 498
Minneapolis Meeting, The........ 387
Missing Numbers........ 559

New Forms of Scalers........ 556
Notice........ 211

Obituary—Lyman Wells........ 108
Objectionable Terms........ 332
Only Delayed........ 50
Origin of Medical Science, The........ 211
Our Book Table........50, 104, 211, 334, 441, 614

Patent Bridge Work........ 274
Prehistoric Dentistry........ 46
Proposed International Dental Congress, The........ 500

Sewill's Dental Caries........ 273
Sneaking into Dentistry........ 159

To Junior Dentists........551, 609, 664
Twenty-fifth Meeting of the A. D. A., The........ 441

Vulcanite Plates........ 103
Vulcanizing........ 272

Which Way........ 382
Wonderful Piece of Mechanism, A........ 611
Worth Attention........ 274

### CURRENT NEWS AND OPINION.

About Exposed Dental Pulps........ 560
Administration of Nitrous Oxide........ 112
Amalgam Solvents........393, 447
American Academy of Dental Science........ 678
American Dental Association........289, 390, 449
American Dental Society of Europe........ 337
American Medical Association........ 168
Anæsthetics—Good, Bad and Indifferent........ 507
Annals of Surgery........ 56
Annual Dinner: Alumni Association of the New York College of Dentistry, 281
Askings and Answers........58, 114, 170, 226, 282, 338, 394
Assimilation of Iron........ 503

California........ 277
Capping Exposed Pulps........ 166
Change of Location........ 563
Chicago College of Dental Surgery........ 280
Chicago Dental Society........ 280
Chloroform and the Fifth Pair of Nerves........ 169
Chloroform *vs.* Ether........ 111
Circular Letter to Members of Section VII. "Physiology and Etiology," of the American Dental Association........ 675
Concerning the Syphilitic Origin of Rachitis........ 452
Copper and Lead in Food........ 486
Connecticut Valley Dental Society........ 564, 677
Connecticut Valley and Massachusetts Dental Societies........ 337
Consolidation........ 563
Correction, A........ 281

Dental Assistants........ 167
Dental Education in Great Britain........ 560
Dental Furnace........ 166
Dental Hygiene........ 508
Dental Journals Wanted........ 279
Dental Law of Minnesota........ 336
Dental Legislation in Dakota........ 225
Delinquents........ 622
Dental Novelist, A........ 508
Dental School in Austria........ 168
Dental Society of the State of New York........ 278
Denture in the Stomach, A........ 451
Dinner to Mr. Charles Tomes........ 109
Doctor Swasey's Economic Disk Moulds........ 57
Donaldson's Nerve Broaches........ 57

Eighth District Dental Society........ 225
Emendatory........ 164
English Advertisement, An........ 110
Errata........ 279
Excellent Instrument, An........ 622
Expansion of Zinc........ 122
Extirpation of the Larynx........ 451
Extraction of Teeth........ 169
Evidences of Prosperity........ 622

Ferments........ 506
Fifth and Sixth District Dental Societies........ 564
Fifth District Dental Society........ 224
First District Dental Society........ 618
Foreign Bodies in the Antrum of Highmore........ 620
From Russia........ 163

German Dental Fees........ 278
Gold Medal Award........ 110
Good Investment, A........ 677
Gutta-Percha Cappings........ 449

Honors Honestly Won........ 508
How to Keep Cider Sweet........ 111
Hydro-Chlorate of Çocaine........ 220

Illinois State Dental Society........ 221
Ingenious........ 449
International Medical Congress, The........ 393
International Tooth Crown Company, The........ 225

Jury Duty........ 16

Kansas State Dental Association.... 335
Kansas State Dental Law...... 223
Kentucky State Dental Association. 335

Law of Finding, The. 507
Listerine. 55

Married. Wilkerson–Bowie. 166
Massachusetts Dental Society. 678
Massachusetts and Connecticut Valley Dental Societies. 278
Medical Congress of 1887, The. 224
Menthol. 162
Mike and the Doctor. 450
Minnesota. 622
Mississippi Valley Association of Dental Surgeons. 109
Missouri State Dental Association. 336
Mrs. Lucretia Brackett. 448

National Association of Dental Examiners. 168, 450
National Association of Dental Faculties. 337
Nebraska State Dental Society. 225
New England Dental Society. 621
New Jersey State Dental Society. 392, 621
New Mecca on the Maine Coast, A. 508
New York College of Dentistry. 222
New York Odontological Society. 56
Noblesse Oblige. 165
Notice to Subscribers. 281

Obituary—J. G. Ambler. 275
Obituary—John M. Riggs. 674
Obituary—Louis Elsberg. 160
Odontological Dinner. 222
Ohio State Dental Society, The. 564
Our Rules. 113

Peculiar Case, A. 505
Pennsylvania State Dental Society. 391
Pennsylvania State Society Reports. 57
Personal. 506
Plain Truth Well Expressed, A. 622
Preserving Tooth Pulps. 221
Prescriptions Useful for Nervousness. 112

Remittances. 57

Second District Dental Society. 224
Sensitive Dentine. 450
Seventh and Eighth District Dental Societies. 564
Seventh District Dental Society. 225

Sixth District Dental Society.... 392
Southern Dental Association.... 165
Specimen, A.... 111, 505

Tennessee Dental Association.... 112
To Advertisers.... 393
Tooth Crown Patents, The.... 276
To Avoid Nausea.... 111
To Subscribers.... 392
Treatise on Diphtheria, A.... 677
Treatment of Cholera.... 163

University of Maryland.... 504
University of Maryland—Dental Department.... 222

Wanted.... 57
Want Supplied, A.... 678
Welcome Home, A.... 112
What is Dental Surgery.... 388
Williams Engine Gold Condenser.... 448
Wooden Toothpick, The.... 336

### The Independent Practitioner—Vol. VI.

### PUBLISHED BY DENTISTS FOR DENTISTS.

### Prospectus.

THIS JOURNAL is published by an association of professional men who have no ulterior objects other than to afford to the profession an independent, outspoken Journal, which shall be the exponent of higher aims and a better practice.

Having no connection with any school, clique, or advertising firm, it will be impartial in its judgment of professional matters, and its appeals for support are directed to those who believe that a liberal profession should have an independent literature.

While not neglecting practical details, it will pay especial attention to those oral diseases that have usually been under the care of the general practitioner, but which more properly belong to the specialty of dentistry.

Its pages will be open to any member of the profession who has anything of interest to communicate, and it invites a free expression of views on all subjects medical or dental.

As each of the gentlemen forming the association is practically a member of the editorial corps, it will have abundant opportunity for collecting all the latest professional news and intelligence.

It will have a body of regular contributors, selected from the very best writers in the profession, and will thus be certain of an abundance of interesting original matter.

It will pay especial attention to reports of the meetings of dental societies, and its aim will be to publish an epitome of all important discussions of professional matters.

It will contain original abstracts and translations of all that is of moment in the foreign journals.

Its profits, if any, will be devoted to its improvement by adding needed illustrations, paying for valuable original communications, and enlarging its size whenever this becomes advisable.

To the accomplishment of the work thus outlined (necessarily relying upon the hearty approval of an appreciative profession), each of the publishers of the INDEPENDENT PRACTITIONER has pledged his best efforts.

Editorial communications should be addressed to DR. W. C. BARRETT, No. 208 Franklin St., Buffalo, N. Y. Send all business letters to DR. WILLIAM CARR, 35 West Forty-Sixth Street, New York.

| | | |
|---|---|---|
| ABBOTT, FRANK ...New York. | CARR, WILLIAM..New York. | JARVIE, WM. Jr..Brooklyn. |
| BARRETT, W. C..........Buffalo. | FRANCIS, C. E....New York. | NORTHROP, A. L.New York. |
| BODECKER, C F. W..New York. | HILL, O. E...........Brooklyn. | PALMER, S. B......Syracuse. |

W. D. MILLER, Berlin, Germany.

### The Independent Practitioner—Vol. VI.

### PUBLISHED BY DENTISTS FOR DENTISTS.

### Prospectus.

THIS JOURNAL is published by an association of professional men who have no ulterior objects other than to afford to the profession an independent, outspoken Journal, which shall be the exponent of higher aims and a better practice.

Having no connection with any school, clique, or advertising firm, it will be impartial in its judgment of professional matters, and its appeals for support are directed to those who believe that a liberal profession should have an independent literature.

While not neglecting practical details, it will pay especial attention to those oral diseases that have usually been under the care of the general practitioner, but which more properly belong to the specialty of dentistry.

Its pages will be open to any member of the profession who has anything of interest to communicate, and it invites a free expression of views on all subjects medical or dental.

As each of the gentlemen forming the association is practically a member of the editorial corps, it will have abundant opportunity for collecting all the latest professional news and intelligence.

It will have a body of regular contributors, selected from the very best writers in the profession, and will thus be certain of an abundance of interesting original matter.

It will pay especial attention to reports of the meetings of dental societies, and its aim will be to publish an epitome of all important discussions of professional matters.

It will contain original abstracts and translations of all that is of moment in the foreign journals.

Its profits, if any, will be devoted to its improvement by adding needed illustrations, paying for valuable original communications, and enlarging its size whenever this becomes advisable.

To the accomplishment of the work thus outlined (necessarily relying upon the hearty approval of an appreciative profession), each of the publishers of the INDEPENDENT PRACTITIONER has pledged his best efforts.

Editorial communications should be addressed to DR. W. C. BARRETT, No. 208 Franklin St., Buffalo, N. Y. Send all business letters to DR. WILLIAM CARR, 35 West Forty-Sixth Street, New York.

| | | |
|---|---|---|
| ABBOTT, FRANK....New York. | CARR, WILLIAM..New York. | JARVIE, WM. Jr..Brooklyn. |
| BARRETT, W. C..........Buffalo. | FRANCIS, C. E....New York. | NORTHROP, A. L.New York. |
| BODECKER, C. F. W..New York. | HILL, O. E..........Brooklyn. | PALMER, S. B......Syracuse. |

W. D. MILLER, Berlin, Germany.

